# The Outer Membrane Proteins and Their Synergy Triggered the Protective Effects against Pathogenic *Escherichia coli*

**DOI:** 10.3390/microorganisms10050982

**Published:** 2022-05-08

**Authors:** Guihong Pen, Na Yang, Da Teng, Ya Hao, Ruoyu Mao, Jianhua Wang

**Affiliations:** 1 Gene Engineering Laboratory, Feed Research Institute, Chinese Academy of Agricultural Sciences, Beijing 100081, China; pgh1316191@163.com (G.P.); nana_891230@126.com (N.Y.); tengda@caas.cn (D.T.); haoya@caas.cn (Y.H.); 2 Innovative Team of Antimicrobial Peptides and Alternatives to Antibiotics, Feed Research Institute, Chinese Academy of Agricultural Sciences, Beijing 100081, China; 3 Key Laboratory of Feed Biotechnology, Ministry of Agriculture and Rural Affairs, Beijing 100081, China

**Keywords:** *Escherichia coli*, outer membrane protein, combination immunization, immune response

## Abstract

Colibacillosis caused by pathogenic *Escherichia coli* (*E. coli*) is one of the most serious infectious diseases, causing an extensive burden on animal husbandry and the human healthcare system. Vaccination is one of the ideal ways to prevent *E. coli* infection. In this work, recombinant outer membrane protein A (rOmpA), outer membrane protein C (rOmpC) and BamA (rBamA) from *E. coli* O78 (CVCC CAU0768) were expressed in a prokaryotic expression system with the concentration of 1–2 mg/mL after purification. Considerable immune responses could be triggered in mice that were immunized with these recombinant proteins, high antibody titers, high total IgG level and various antibody isotypes were detected in antisera after booster immunizations. Moreover, mice immunized with several recombinant proteins in combination showed a higher survival rate with the challenge of homologous strain *E. coli* O78 and a more significant cross-protection effect against heterologous strain *E. coli* O157:H7 (CICC 21530) in vivo than those of immunized alone. The antisera from immunized mice showed high affinity to multiple strains of *Escherichia*, *Shigella* and *Salmonella* in vitro, indicating that recombinant outer membrane proteins from *E. coli* O78 had the potential to be developed into universal antigenic substances against not only *E. coli* but also a variety of Gram-negative bacteria. rOmpA was considered as the most immunogenic protein in this work and the combination of different proteins could further enhance the immune response of immunized mice, which provided the reference for the construction of novel antigens with higher efficiency.

## 1. Introduction

There are two main pathogenic *E. coli* clinical pathotypes, including intestinal pathogenic *E. coli* (IPEC) and extraintestinal pathogenic *E. coli* (ExPEC), in which IPEC is the primary cause of diarrhea- and intestinal-related diseases, while ExPEC is the major cause of extraintestinal infections including urinary tract infections, neonatal sepsis, meningitis, etc. [1,2,3,4]. IPEC strains such as enterotoxigenic *E. coli* (ETEC), enteroaggregative *E. coli* (EAEC), enterohemorrhagic *E. coli* (EHEC), etc., primarily give rise to intestinal-related diseases and almost do not cause extraintestinal infection in the healthy hosts except in the developing world [2]. EHEC can lead to diarrhea, hemorrhagic colitis and hemolytic uremic syndrome, of which serotype O157:H7 plays a major role [5,6]. ExPEC strains such as avian pathogenic *E. coli* (APEC), septicemic pathogenic *E. coli* (SPEC) and uropathogenic *E. coli* (UPEC), etc., have a unique ability to invade various organs or tissues outside the gastrointestinal tract of hosts, resulting in various life-threatening diseases [3]. Reports showed that about 80% of APEC infections are caused by serotypes O78, O1 and O2 [7], leading to great economic losses to the poultry industry worldwide [8,9,10]. Antibiotics have made a great contribution to the treatment of *E. coli* infection, but the consequences of which cannot be ignored [11]. The rates of antibiotic resistance in *E. coli* are very high in China, India and the Middle East [2]. In addition, serotyping of *E. coli* is complex, with 173 kinds of O antigen, 80 kinds of K antigen, and 56 kinds of H antigen, which can be found in many possible combinations in nature, eventually forming 50,000–100,000 or more serotypes [12]. Although there are more and more studies and reports about vaccines for *E. coli* based on serotype, there will be a tremendous amount of work to do.

The academic, technical and industrial advantages of subunit vaccines are: moving the gateway of epidemic prevention ahead, mastering the risk of infection under control, delaying the mutation of pathogens, extending the validity span of vaccines, preventing the epidemics effectively at optimal ratio of cost performance in terms of time, results, scale and other input in whole. Therefore, subunit vaccines have become important candidate vaccine systems for rapid response to the spread of emergent diseases and pandemics. Outer membrane proteins (OMPs) broadly exist in Gram-negative bacteria and belong to the transmembrane β-barrel family. OMPs are not only involved in the processes of protein secretion, adhesion, virulence and biofilm formation of bacteria but also potential drug and vaccine targets as surface-exposed molecules [13]. As a consequence, the OMPs subunit vaccine has become an important research target for the treatment of Gram-negative bacteria. It has been reported that rOmpA, rOmpC and rBamA from *E. coli* CVCC 1515 exhibited a high immunogenicity, a strong humoral immune response could be detected in serum from mice that were immunized with these OMPs and Freund’s adjuvant [14,15,16]. Pore et al. found that rOmpA from *Shigella flexneri* 2a could evoke protective immunity in mice, which involved the participation of both the humoral and cellular immune responses [17]. Li et al. indicated that rOmpF could not only induce chicken to generate a high level of antibody but also induce chicken to produce a strong cell-mediated immune response, showing a high protective effect against *Salmonella enteritidis* infection [18]. Zhang et al. indicated that rOmpA from *Proteus mirabilis* as an antigen could enhance the immune response in chickens [19]. Zhang et al. found that OMPs of *Klebsiella pneumoniae* as immunogen could stimulate mice to produce significant antigen-specific IgG such as IgG2a and IgG1, which could be developed as serotype-independent and multivalent vaccine against infection caused by *K. pneumoniae* [20]. Duan et al. found that purified rOmpA from *Edwardsiella anguillarum* as an immunogen could significantly improve the specific and non-specific immune response in Japanese eels, thus promoting the resistance of eel to *E. anguillarum* infection. They indicated that it may be a feasible strategy for preparing practical aquatic vaccines [21]. Diao et al. indicated that the immunogenic surface recombinant protein rOmpC from *Aeromonas salmonicida* subsp. *masoucida* (ASM) could elicit a strong humoral immune response in rainbow trout and then provide a significant protective effect against ASM infection [22]. The above studies suggest that OMPs from different Gram-negative bacteria are ideal antigens in mice, chickens or fish, but not all OMPs are effective; more work is needed to screen out proteins with higher immunogenicity. In addition, most of the current studies only immunized a single OMP from Gram-negative bacteria in animals to characterize its anti-infection effect on the homologous strain. The lack of studies on both the anti-infection effect on the heterologous strain and the horizontal comparison among different proteins has made it difficult to find the most characteristic antigen.

The *E. coli* O78 (CVCC CAU0768), which belongs to extraintestinal APEC with various virulence factors, was isolated from the faeces of diarrhea avian. The OMPs of this strain have not been revealed. Therefore, to search for more characteristic antigenic proteins, the OMPs including OmpA, OmpC and BamA from clinical *E. coli* O78 were cloned and expressed in this work. The efficacy of these recombinant proteins as subunit vaccines was evaluated in mice by immunized alone or in combination, the protective effect against both homologous strain and heterologous strain in vivo and in vitro was validated, and the most characteristic antigenic protein was determined by horizontal comparisons with different proteins.

## 2. Materials and Methods

### 2.1. Bacterial Strains and Plasmids

*E. coli* O78 (CVCC CAU0768) was donated by Professor Aike Li from the Academy of National Food and Strategic Reserves Administration. *Shigella* *sonnei* CVCC 3926 was donated by Professor Bo Shi from the Feed Research Institute of Chinese Academy of Agricultural Sciences. *Salmonella enteritidis* CVCC 3377 were bought from the China Veterinary Culture Collection Center (CVCC) (Beijing, China). *Sh. flexneri* CMCC (B) 51571 was purchased from the National Center for Medical Culture Collection (CMCC) (Beijing, China). *Pseudomonas aeruginosa* CICC 21630, *P. aeruginosa* CICC 10419 and *E. coli* O157 (CICC 21530) were purchased from the China Center of Industrial Culture Collection (CICC) (Beijing, China). *E. coli* competent strains of BL21 (DE3) and DH5α were purchased from TransGen Biotech Co., Ltd. (Beijing, China). The vector of pET-28a (+) was purchased from Novagen (Madison, WI, USA) and pMD^TM^ 19-T Simple was purchased from TaKaRa Biotechnology (Dalian, China).

### 2.2. Characterization of E. coli O78

Classical virulence factors of APEC were amplified by PCR to characterize *E. coli* O78 (CVCC CAU0768). Eight virulence genes including *irp2*, *iss*, *fimC*, *iroN*, *mat*, *iebB*, *iucD* and *fyuA* were detected in *E. coli* O78. The primers, *irp2*-F: 5′-CTGATGAACTCACTCGCTATCC-3′, *irp2*-R: 5′-AGCATCTCCTGGCTCTGCTC-3′, *iss*-F: 5′-ATCACATAGGATTCTGCCG-3′, *iss*-R: 5′-CAGCGGAGTATAGATGCCA-3′, *fimC*-F: 5′-GCCGATGGTGTAAAGGATGG-3′, *fimC*-R: 5′-GGGTAAGTGCGCCATAATCA-3′, *iroN*-F: 5′-CCTCCGACGATGATAATGACG-3′, *iroN*-R: 5′-GATACCATTATGCGTAATGCC-3′, *mat*-F: 5′-CGACCTGGTCAGCAACAGCC-3′, *mat*-R: 5′-TCCACGCCCACATTCAGTGT-3′, *iebB*-F: 5′-GTTCTCACTCAGCCAGAACG-3′, *iebB*-R: 5′-CATCCAGCACTTCCAGATAAC-3′, *iucD*-F: 5′-GAAGCATATGACACAATCCTG-3′, *iucD*-R: 5′-CAGAGTGAAGTCATCACGCAC-3′, *fyuA*-F: 5′-ATGTGAAACTGCGTCTGGCG-3′, *fyuA*-R: 5′-CGCAGTAGGCACGATGTTGT-3′, were obtained to amplify the genes [23,24,25,26].

### 2.3. Homological Analysis of the OmpA, OmpC and BamA

Gene sequences of OmpA, OmpC and BamA from *E. coli* O78 (CVCC CAU0768) were obtained by PCR amplification and amino acid sequences were translated. The amino acid sequences were blasted by online database UniProt [27] (https://www.uniprot.org/blast/, accessed on 26 November 2021) with the below parameters: Matrix BLOSUM-62, E-Threshold 10, no filtering, and the number of feedback alignments was 50. Based on similarities, the blasted data of *Escherichia*, *Shigella*, *Salmonella* and *Klebsiella* from the top 50 sequences were aligned, the phylogeny trees were constructed by MEGA (version 11, Kim Dotcom, Auckland, New Zealand) with the method of Neighbor-Joining and the number of bootstrap replications was 1000 [28,29].

### 2.4. Cloning of the ompA, ompC and bamA Gene

Genomic DNA of *E. coli* O78 (CVCC CAU0768) was extracted by Bacteria DNA Kit (Tiangen Biotech Co., Ltd., Beijing, China). The primers with the respective restriction sites, including rOmpA F-*Eco*RI: 5′-GAATTCGCTCCGAAAGATAACACCTGGTACAC-3′, rOmpA R-*Not*I: 5′-GCGGCCGCAGCTTGCGGTTGAGTTACTACGTC-3′, rOmpC F-*Bam*HI, 5′-CGGGATCCGCTGAAGTTTACAACAAAGACG-3′, rOmpC R-*Xho*I, 5′-CCCTCGAGTTAGAACTGGTAAACCAGACCC-3′, rBamA F-*Eco*RI: 5′-GAATTCAATTGGTTAGGTACAGGTTATGC-3′, rBamA R-*Not*I: 5′-GCGGCCGCCCAGGTTTTGCCGATGTTGAACT-3′, were obtained and used for amplifying the genes by PCR according to the previous method with some modifications [14,15,16]. The target fragments were cloned into the pMD^TM^ 19-T Simple vector and transformed into *E. coli* DH5α. The positive recombinant plasmids were isolated by the Plasmid Extraction Kit (Tiangen Biotech Co., Ltd., Beijing, China) and digested with the corresponding enzymes. After digestion, target DNA fragments were ligated into the expression vector pET-28a (+) to construct the plasmids pET28a-ompA/ompC/bamA. The recombinant plasmids with purpose fragments were further transformed into the expression strain *E. coli* BL21 (DE3); positive strains were screened by PCR amplification and sequencing of DNA.

### 2.5. Expression and Purification of the rOmpA, rOmpC and rBamA Protein

The recombinant proteins with 6× His-tag were expressed in BL21 (DE3) induced by Isopropyl β-D-1-Thiogalactopyranoside (IPTG) [30]. In simple terms, the positive target strains were cultured with 250 rpm at 37 °C in LB medium, when the optical density at 600 nm (OD_600nm_) ranged from 0.40 to 0.60, the IPTG was added into the medium with the final concentration of 1 mM. Then, the strains were continually cultured under the same condition for 6–8 h. The finished induced cells were obtained by centrifuging with 5000× *g* at 4 °C for 30 min, the supernatant was removed, and the precipitate was resuspended in 50 mM Tris–HCl lysis buffer, (pH 7.9, involving 5 mg of lysozyme/gram of cell paste) with a volume of 8 mL/g wet weight. To disrupt the cells adequately, the solution was sonicated on ice for 20 min by a probe ultrasonicator (SCIENTZ, Ningbo Science Biotechnol Co., Ltd., Ningbo, China) with a frequency of 10 s off and 5 s on. Then, the solution was centrifuged with 14,500× *g* at 4 °C for 20 min, the supernatant was removed, and the insoluble inclusion bodies (IBs) were obtained. Next, the precipitated IBs were dissolved by 10 mM Tris-HCl denaturing buffer (pH 7.5, involving 8 M urea). The denatured solution was centrifuged with 14,500× *g* at 4 °C for 20 min, the supernatant was collected, an equal volume of 20 mM Tris–HCl refolding buffer (pH 7.9, involving 5% (*v*/*v*) lauryl dimethyl amine oxide (LDAO) and 1 M NaCl) was added dropwise with rapid stirring to refold the recombinant proteins [31].

The refolded recombinant proteins were purified according to the previous methods with some modifications [14]. Firstly, the His Bind column of Ni^2+^-NTA affinity chromatography (AKTAxpress, Fairfield, CT, USA) was pre-equilibrated with 20 mM Tris–HCl equilibration buffer (pH 7.9, involving 0.1% (*v*/*v*) LDAO, 500 mM NaCl and 0.04 M imidazole). Next, the refolded recombinant protein solution was loaded into the column and then washed with 20 mM Tris–HCl elution buffer (pH 7.4, involving 0.1% (*v*/*v*) LDAO, 500 mM NaCl and 0.5 M imidazole), the recombinant proteins were eluted and collected. Finally, the collected recombinant proteins were desalted by dialyzing with 20 mM Tris–HCl buffer (pH 7.4, involving 0.1% (*v*/*v*) LDAO and 0.15 M NaCl). The concentration and purity of recombinant proteins were analyzed by the BCA Protein Assay Kit (Tiangen Biotech Co., Ltd., Beijing, China) and Gel-Pro Analyzer™ version 6.3 (Bio-Rad, CA, USA), respectively.

### 2.6. Mouse Immunization

The immunization programs were conducted by the previous method with some modifications [14,28]. The SPF BALB/c mice were purchased from Vital River Laboratory Animal Technology Co., Ltd. (Beijing, China). A total of 135 female SPF BALB/c mice aged from 6–8 weeks were divided into 9 groups and immunized with the purified rOmpA-adjuvant, rOmpC-adjuvant, rBamA-adjuvant, rOmpA + rOmpC-adjuvant, rOmpA + rBamA-adjuvant, rBamA + rOmpC-adjuvant, rOmpA + rBamA + rOmpC-adjuvant, PBS-adjuvant, respectively. One group of mice was not immunized and raised under the same conditions as a negative control. The purified proteins were diluted with sterile PBS to a final concentration of 0.5 mg/mL and then mixed with complete Freund’s adjuvant in a volume ratio of 1:1 for the primary immunization and incomplete Freund’s adjuvant for the booster immunizations. A volume of 100 μL/mouse antigen mixture was vaccinated by hypodermic injection for the primary immunization on day 0 and intraperitoneal injection for the booster immunizations on day 21 and 35. All mice were monitored daily and housed reasonably in the cages with ventilation (Suhang Technology Equipment Co., Ltd., Suzhou, China) which were changed weekly. Blood was collected on day 5, day 26 and day 40 through the tail vein of mice. Sera were divided into small portions and stored at −80 °C until used.

### 2.7. Antibody Titer and Antibody Isotypes Detection by iELISA

Antibody titer and antibody isotypes were detected by protein iELISA. Firstly, the 96-well ELISA plates were coated with 100 μL of each protein (rOmpA, rOmpC, rBamA and an equimolar mix rOmpA + rOmpC, rOmpA + rBamA, rOmpC + rBamA, rOmpA + rOmpC + rBamA) at a final concentration of 2 µg/mL in coating buffer (pH 9.6, 35 mM sodium bicarbonate, 15 mM sodium carbonate) and incubated at 4 °C overnight. Then, the plates were washed by PBST (10 mM PBS, involving 0.05% Tween 20) for 4 times. Next, 5% BSA which dissolved in PBST with a volume of 200 μL was used to block the plates at 37 °C for 4 h. After washing, a volume of 100 μL serially diluted (from 1:1000) mouse serum was added and the plates were incubated at 37 °C for 1.5 h. After washing, a volume of 100 μL Horseradish Peroxidase (HRP) conjugated goat anti-mouse total IgG, IgG1, IgG2a, IgG2b, IgG3, IgA and IgM which diluted at 1:5000 was added to plates and incubated at 37 °C for 30 min. After washing, 3,3′,5,5′-tetramethylbenzidine (TMB) with 100 μL/well was added and the plates were incubated in the dark at 37 °C for 25 min. Finally, the reaction was terminated by adding 100 μL H_2_SO_4_ with a concentration of 2 mol/L. The OD value of each well was measured by a spectrophotometer (TECAN, Grödig, Austria) at 450 nm with the reference wavelength of 630 nm. Endpoint titers were calculated as the reciprocal of the last serum dilution that gave a value 2.1 times higher than non-immunized negative control with a minimum value of 0.05 [32,33].

### 2.8. Cross-Protection Property Detected by iELISA

The cross-reaction properties of antisera against different Gram-negative bacteria including *E. coli* O78 (CVCC CAU0768), *E. coli* O157 (CICC 21530), *Sh.* *sonnei* CVCC 3926, *Sh. flexneri* strain CMCC (B) 51571, *S. pullorum* CVCC 1789, *S. enteritidis* CVCC 3377, *P. aeruginosa* CICC 21630 and *P. aeruginosa* CICC 10419 were measured by the whole-cell iELISA. A volume of 150 μL/well stationary liquid (0.1 M NaHCO_3_, involving 5% glutaraldehyde) was added to pretreat the plate at 37 °C for 2 h. After washing, a volume of 100 μL solution with 10^7^ CFU bacteria was added to each well and aired at 37 °C [28]. The next steps were the same as for protein iELISA above.

### 2.9. Double-Immunodiffusion Assay

For analyzing the level of total IgG, a double-immunodiffusion assay was applied as described previously with some modifications [34]. Briefly, agarose powder was dissolved with sterile 0.9% NaCl solution in a glass beaker to reach a final concentration of 1%. The mixture solution was heated at 100 °C about 5 min to obtain a homogeneous solution, which was moved to a 56 °C water bath for heat preservation. A volume of 10 mL melted solution at 56 °C was placed in a small beaker, and the goat anti-mouse IgG antibody was added with the concentration determined by the pre-test (final concentration 250 μg/mL). After thorough mixing, the solution with antibody was gently poured to plastic plates which were placed on a horizontal surface and kept for 30 min. When the solution solidified into a flat gel, a series of holes with an aperture of 3–4 mm and a hole distance of 10 mm were punched with a puncher. A volume of 10 μL standard mouse IgG with different concentrations (0.75, 0.5, 0.25, 0.125 and 0.0625 mg/mL) were accurately added into the hole with micropipette. Likewise, the serum to be tested was diluted into 1:2 with 0.9% NaCl solution and added to the hole. Then, the plates were covered and kept in a humidified chamber that contained wet cotton and placed on a horizontal surface at 37 °C for 24 h. The diameter of the precipitation ring was measured and recorded. With 5 gradient concentrations of standard mouse IgG as independent variables and the diameter of the precipitation ring as dependent variables, a regression equation was established through linear regression and correlation to calculate the IgG content in immune serum. All the experiments were performed in triplicate.

### 2.10. Opsonophagocytosis Assay

The serum on day 40 was used for the Opsonophagocytosis assay as described previously with some modifications [14,35]. Briefly, a volume of 10 μL antisera was incubated with 90 μL of mid-log phase *E. coli* O78 (CVCC CAU0768) cells with the concentration of 4 × 10^4^ CFU/mL at 37 °C for 30 min. Then, a volume of 100 μL of phagocyte suspension which was obtained from peritoneal fluid of mice with the concentration of 4 × 10^6^ cells/mL was added into the mixed solution with antisera and bacteria, and then incubated at 37 °C for 1 h. After that, sterile water was added to the mixture to lyse the phagocytes, then the mixture solution was diluted in serial, and available bacteria was evaluated by plate count. The formula (1 − (No. of CFU recovered in the presence of phagocytes/No. of CFU recovered in the absence of phagocytes) × 100) was used to calculate the bacterial killing rate [14].

### 2.11. Challenge Assay

The in vivo immune protection induced by recombinant protein in mice was characterized by the challenge with homologous and heterologous strains. An absolute lethal dose (LD_100_, 3 × 10^7^ CFU/mice) of ExPEC *E. coli* O78 (CVCC CAU0768) in mice was determined in advance. On day 49, 10 mice of each group were intraperitoneally injected with 2 × LD_100_ mid-log phase *E. coli* to evaluate the effectiveness of recombinant proteins against a homologous strain. Mortality was recorded for 7 days. The remaining 5 mice of each group were intragastrically administrated with 10^10^ CFU mid-log phase IPEC *E. coli* O157 (CICC 21530) after fasting for 12 h to identify the protective effect of recombinant proteins on the heterologous strain. Fecal samples were collected every day during the 7-day observation period, and the fecal shedding of bacteria was determined by plate colony count [28].

## 3. Result

### 3.1. Characterization of E. coli O78

**Figure 1:**
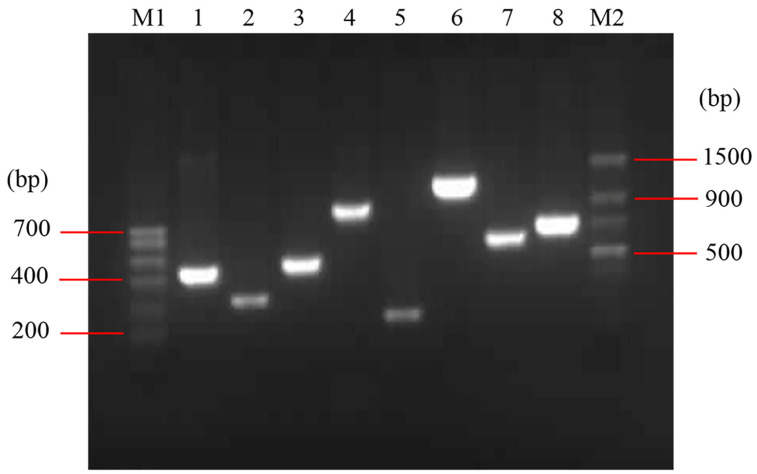
Identification of virulence factors in *E. coli* O78 (CVCC CAU0768). M1: Trans DNA Marker I; M2: Trans DNA Marker II; lane 1–8: *irp2*, *iss*, *fimC*, *iroN*, *mat*, *iebB*, *iucD* and *fyuA*, respectively.

The detected virulence factors of *E. coli* O78 (CVCC CAU0768) were related to adhesion (*mat*, *fimC*), iron acquisition system (*iroN*, *fyuA*, *irp2* and *iucD*), invasion (*ibeB*) and protectins/serum resistance (*iss*) (Figure 1). The *mat* is temperature-regulated fimbriae and associated with meningitis, *fimC* is responsible for adherence and colonization of epithelial cells. *iroN* is a catecholate siderophore (salmochelin) receptor, *fyuA* is a ferric yersiniabactin receptor, *irp2* is responsible for iron repressible protein yersiniabactin synthesis, and *iucD* is related to the mediation of aerobactin synthesis. The *iss* promotes bacterium protection, which mainly relates to the action of the complement system, and *ibeB* is involved in APEC invasion and pathogenicity [23,24,25,26]. The result indicated that *E. coli* O78 (CVCC CAU0768) used in this work is a virulent strain with multiple virulence factors, which is a typical representative in APEC.

### 3.2. Homological Analysis of the OmpA, OmpC and BamA

Blasting was conducted by the UniProt database, the top 50 sequences with the highest similarity to OmpA, OmpC and BamA of *E. coli* O78 (CVCC CAU0768) were mostly from *Escherichia*, *Shigella*, *Salmonella* and *Klebsiella*. The OmpA shares 88.4–98.8% identity with other *Escherichia*, and shares 94.5–96.1%, 91.5–92.1% and 83.0–86.7% identity with *Shigella*, *Salmonella* and *Klebsiella*, respectively. The OmpC shares 91–97.7% identity with other *Escherichia*, and shares 92.4–94.3%, 79.2–80% and 79.2–81.7% identity with *Shigella*, *Salmonella* and *Klebsiella*, respectively. The BamA shares 86.5–100% identity with other *Escherichia*, and shares 100%, 92.3–93.7% and 85.5–87.4% identity with *Shigella*, *Salmonella* and *Klebsiella*, respectively. The results of blast and phylogenetic analysis showed that OmpA, OmpC and BamA from *E. coli* O78 (CVCC CAU0768) were highly homologous to those in *Escherichia*, *Shigella*, *Salmonella* and *Klebsiella* (Figure S1), indicating that these OMPs have potential to be developed into universal antigens against a variety of Gram-negative bacteria.

### 3.3. Cloning, Expression, and Purification of the Recombinant Proteins

**Figure 2:**
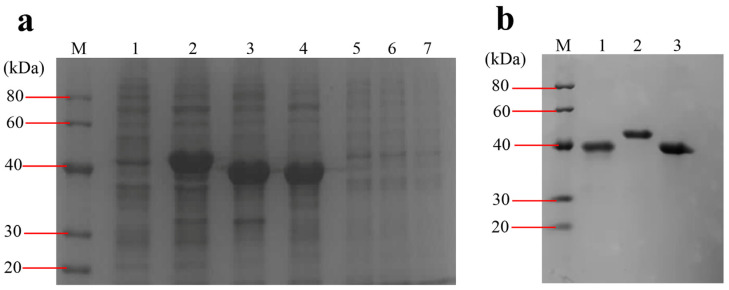
Expression and purification of the OMPs. (**a**) Expression of the OMPs. Lane M, protein marker (20–40 kDa); lane 1, total proteins of BL21 harboring pET28a vector induced by IPTG; lane 2–4, total proteins of BL21 harboring pET28a-bamA, pET28a-ompA, pET28a-ompC induced by IPTG, respectively; lanes 5–7, total proteins of BL21 harboring pET28a-bamA, pET28a-ompA, pET28a-ompC without being induced by IPTG, respectively. (**b**) Purified OMPs. Lane 1–3, eluent of the purified rOmpA, rBamA, rOmpC, respectively.

The DNA fragments of *ompA* (GenBank Accession No. OM350111), *bamA* (GenBank Accession No. OM365872) and *ompC* (GenBank Accession No. OM365873) with the size of 975 bp, 1089 bp and 1026 bp, respectively, were amplified from *E. coli* O78 (CVCC CAU0768) DNA genome. The digestion fragments were cloned into the pET28a expression vector and the recombinant proteins were expressed in *E. coli* BL21 (DE3) induced by IPTG. The recombinant proteins are present in the IBs and fused with an N-terminal 6× His tag. The molecular weight of rOmpA, rBamA and rOmpC was approximately 40 kDa, which accorded with their theoretical values (OmpA 35,158.34 Da, OmpC 37,935.18 Da, BamA 40,731.58 Da). After purification, the concentrations of rOmpA, rBamA and rOmpC were 1.122 mg/mL, 1.167 mg/mL and 2.046 mg/mL, respectively (Figure 2). After purification and refolding, the purities of rOmpA, rBamA and rOmpC were 95%, 98% and 97%, respectively.

### 3.4. Immunogenic Property of Recombinant Proteins in Mice

**Figure 3:**
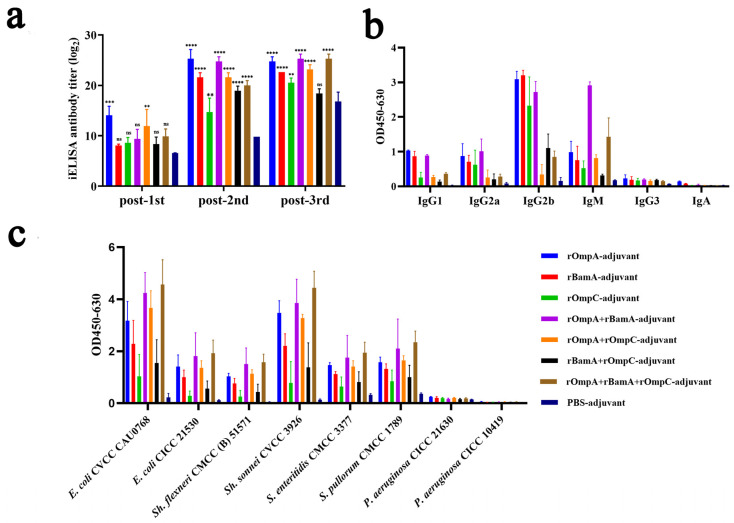
Immune responses in antisera from mice immunized with recombinant proteins. (**a**) Antibody titers of the antisera against recombinant proteins. “**, *** and ****” indicates a significant difference between the immunized group and the PBS-adjuvant group (** *p* ≤ 0.01, *** *p* ≤ 0.001, **** *p* ≤ 0.0001). “ns” indicates that there is no significant difference between the recombinant protein immunized group and the PBS-adjuvant group. (**b**) Antibody isotypes in antisera from immunized mice. The serum dilution was 1:1000. (**c**) Cross-reaction properties of the antisera against different bacteria. The serum dilution was 1:27,000. Data are expressed as the means ± SD (n = 3).

To evaluate the immunogenicity properties of these recombinant proteins, antisera were collected from mice on days 5, 26 and 40 and the specific reactivity with purified recombinant proteins was tested by iELISA. Results revealed that mice immunized with recombinant proteins alone or in combination both exhibited significant immune response; a strong and significant immune response of antisera to the recombinant proteins was detected after the second immunization (Figure 3a). For immunization alone groups, the highest antibody titer reached 1:32,805,000 from the rOmpA-adjuvant group after the third immunization, which was significantly higher than the rOmpC-adjuvant group (1:1,701,000) and rBamA-adjuvant group (1:6,561,000). For the immunization in combination groups, the highest antibody titer reached 1:45,927,000 from both the rOmpA + rBamA-adjuvant group and rOmpA + rBamA + rOmpC-adjuvant group after the third immunization. All recombinant protein immunization groups showed significant differences compared with the PBS-adjuvant group after the second immunization (Figure 3a).

Antibody isotypes in the serum of immunized mice were analyzed by protein iELISA, and the result showed that IgG1, IgG2a, IgG2b, and IgM were detected (Figure 3b); among them, IgG2b exhibited the highest OD value (0.17–3.35), and then in order, IgM (0.27–1.31), IgG2a (0.09–1.28) and IgG1 (0.08–1.05), and IgG3 and IgA were detected from the recombinant protein immunized groups with low OD values (<0.35). For the PBS-adjuvant group, the OD values of above all antibody isotypes were also very low (<0.26). Moreover, the cross-reaction properties of antisera against different Gram-negative bacteria including *Escherichia*, *Shigella*, *Salmonella* and *Pseudomonas* were conducted by whole-cell iELISA assay, among which *Pseudomonas* strains were detected as negative controls because of the low homology to *E. coli* O78 (CVCC CAU0768). The results showed that the antisera from recombinant protein immunized groups with high OD values had a high affinity to *E. coli* O78 (CVCC CAU0768) (0.47–5.43), *E. coli* O157 (CICC 21530) (0.16–2.85), *Sh.* *sonnei* CVCC 3926 (0.26–5.10), *Sh. flexneri* CMCC (B) 51571 (0.10–2.21), *S. pullorum* CVCC 1789 (0.52–2.80), and *S. enteritidis* CVCC 3377 (0.39–2.72) (Figure 3c), while the affinity of antisera to *P. aeruginosa* was low (<0.26). Furthermore, the antisera from the PBS-adjuvant group with low OD values have low affinity to all above strains (<0.39).

In general, rOmpA immunized alone or in combination with other proteins could stimulate mice to produce a higher antibody titer, and the antisera from these groups had the highest affinity capacity to a variety of Gram-negative bacteria, indicating that rOmpA was the most immunogenic outer membrane protein in this work, and the methods of combination immunization could improve the immune response in mice.

### 3.5. Double-Immunodiffusion Assay

**Figure 4:**
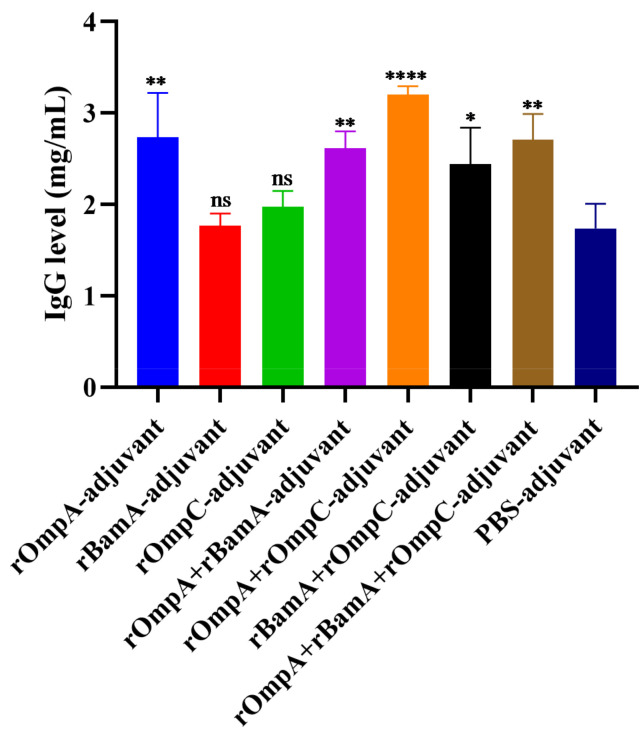
The level of total IgG of the antisera. Data are expressed as the means ± SD (n = 3). “*, ** and ****” indicates a significant difference between the recombinant protein immunized group and the PBS-adjuvant group (* *p* ≤ 0.05, ** *p* ≤ 0.01, **** *p* ≤ 0.0001). “ns” indicates that there is no significant difference between the recombinant protein immunized group and the PBS-adjuvant group.

Quantitative analysis of total IgG levels in antisera after the third immunization was conducted by double-immunodiffusion assay. The level of total IgG in the rOmpA + rOmpC-adjuvant group (3.21 ± 0.09 mg/mL) was the highest among all immunized groups, which was extremely significantly different from the PBS-adjuvant group (1.74 ± 0.27 mg/mL). For immunization alone groups, only the rOmpA-adjuvant group (2.74 ± 0.49 mg/mL) had a significant difference compared with the PBS-adjuvant group (Figure 4). However, there was no significant difference between the PBS-adjuvant group and the rBamA-adjuvant group as well as the rOmpC-adjuvant group. The above results further indicated that rOmpA was the most potential antigenic protein compared with the other two proteins, and the methods of combination immunization could improve the immune response in mice. 

### 3.6. Opsonophagocytosis Assay

**Figure 5:**
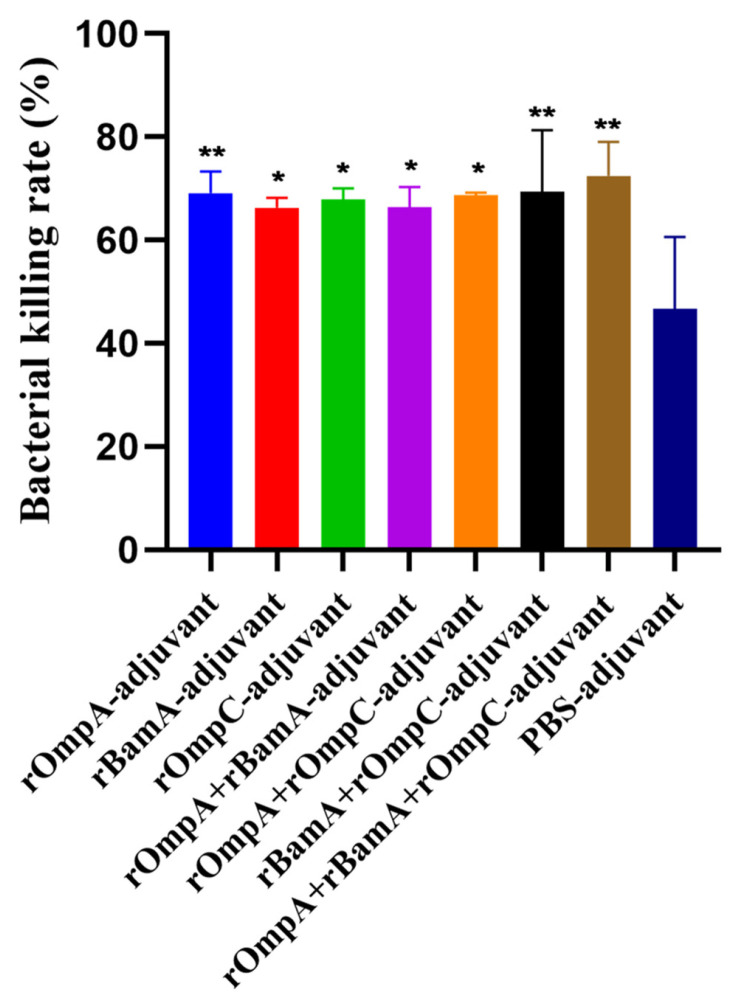
Opsonophagocytosis effect of the antisera in vitro. Colony counts of different groups were determined by plate count. The *E. coli* O78 (CVCC CAU0768) was incubated with antisera of the recombinant proteins immunization group or the PBS immunization group. Data are expressed as means ± SD, n = 3. “* and **” indicates a significant difference between the immunized group and the PBS-adjuvant group (* *p* ≤ 0.05, ** *p* ≤ 0.01).

*E. coli* O78 (CVCC CAU0768) cells were incubated with phagocytes and antisera which were obtained from mice immunized with recombinant proteins or PBS. The bacterial killing rates of rOmpA-adjuvant, rBamA-adjuvant, rOmpC-adjuvant, rOmpA + rBamA-adjuvant, rOmpA + rOmpC-adjuvant, rBamA + rOmpC-adjuvant, rOmpA + rBamA + rOmpC-adjuvant and PBS-adjuvant immunization groups were 69.09 ± 4.19%, 66.28 ± 1.93%, 67.91 ± 2.15%, 66.34 ± 3.96%, 68.73 ± 0.49%, 69.35 ± 11.92%, 72.48 ± 6.56% and 46.73 ± 13.88%, respectively. The bacterial killing rates of recombinant protein immunization groups were all significantly higher than that of the PBS-adjuvant immunization group, the highest bacterial killing rate was from the rOmpA + rBamA + rOmpC-adjuvant group, and the bacterial killing rate of the rOmpA-adjuvant group was higher than those of rBamA-adjuvant group and rOmpC-adjuvant group (Figure 5). The above results showed that the antiserum of mice from the recombinant proteins immunization groups had a more significant opsonophagocytosis effect than that from the PBS-adjuvant group.

### 3.7. Protection Efficacy of Recombinant Proteins against E. coli In Vivo

**Figure 6:**
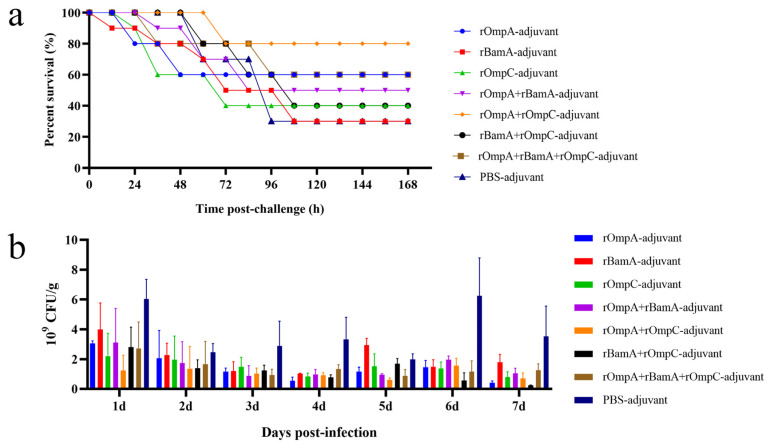
Protection efficacy of the recombinant proteins against *E. coli* in vivo. (**a**) Immunized mice were intraperitoneally injected with mid-log phase *E. coli* O78 (CVCC CAU0768); survival rate was observed for 168 h after the challenge. (**b**) Immunized mice were challenged with mid-log phase *E. coli* O157 (CICC 21530) by gastric tube, and fecal shedding was observed for seven days after the challenge, data are expressed as the means ± SD, n = 3.

On day 49, mice were intraperitoneally injected with 2 × LD_100_ mid-log phase *E. coli* O78 (CVCC CAU0768) to evaluate the immune response induced by recombinant protein against homologous strain infection in vivo. The survival rate: rOmpA-adjuvant group 60%, rOmpC-adjuvant group 40%, rBamA-adjuvant group 30%, rOmpA + rOmpC-adjuvant group 80%, rOmpA + rBamA-adjuvant group 50%, rBamA + rOmpC-adjuvant group 40%, rOmpA + rBamA + rOmpC-adjuvant group 60%, PBS-adjuvant group 30% (Figure 6a). For immunization alone groups, the rOmpA-adjuvant group exhibited the highest survival rate of 60%; for the immunization in combination groups, the rOmpA + rOmpC-adjuvant group showed the highest survival rate of 80%. These results further indicated that rOmpA was the most potential antigenic protein compared with the other two proteins, and the methods of combination immunization could improve the immune response in mice, which was consistent with the result of antisera characterized in vitro.

The antisera of immunized mice displayed cross-reaction to different Gram-negative strains in vitro (Figure 3c), including the IPEC *E. coli* O157 (CICC 21530). Therefore, immunized mice were intragastrically administrated with *E. coli* O157 to further identify the immune response induced by recombinant proteins against heterologous strain infection in vivo. The result of colony counting of fecal samples showed that the recombinant proteins immunization groups had a lower count and a gradual downward trend of *E. coli* in 7 days, while the colony count of the PBS + adjuvant group showed an unstable trend (Figure 6b). The colony counting of the rOmpA + rBamA-adjuvant group, rOmpA + rOmpC-adjuvant group and rOmpA + rBamA + rOmpC-adjuvant group had a significant difference compared with that of the PBS + adjuvant group from the third day after infection (Table S1), indicating that mice immunized with several recombinant proteins in combination could significantly reduce the colonization of heterologous *E. coli* in the intestine and maintain intestinal flora stability.

## 4. Discussion

There have been many studies on outer membrane proteins as subunit vaccines, some of which showed desirable immune effects while some of which were not satisfactory [14,15,16,28,36]. Previous studies showed that OmpA from Gram-negative bacteria was one of the most immunodominant antigens with various properties required for vaccine candidates, such as being involved in bacterial attachment, bacterial conjugation and as receptors for certain bacteriophages [17,37,38]. Therefore, in this work, the recombinant outer membrane proteins including OmpA, BamA and OmpC from highly toxic strain of *E. coli* O78 (CVCC CAU0768) were cloned, analyzed and expressed. The immune effects of these three proteins alone and in combination were evaluated in the mouse model to further identify proteins with more potential.

According to the result of blast on UniProt, the outer membrane proteins were highly conserved in different Gram-negative bacterial strains in the current work. Compared with our previous study [14,15,16], it was found that OmpA, BamA and OmpC from *E. coli* O78 (CVCC CAU0768) not only showed high homology to those in *Escherichia*, *Salmonella* and *Shigella* but also those in *Klebsiella*, among which BamA was the most conservative, and its identity with BamA of 26 strains including *Escherichia coli* and *Shigella* reached 100%. Secondly, OmpA had the highest identity with which of *Escherichia coli* strain K12 and O157:H7, reaching 98.8%. Finally, OmpC had the highest identity with which of *Escherichia coli* O157:H7, which was 97.7% (Figure S1). Previous studies showed that the high conserved property of OMPs made it potential for developing universal vaccines against multiple types of bacteria. Gao et al. showed that OMP VP1243 from *Vibrio parahaemolyticus* possessed immunogenicity and was highly conserved among the major *Vibrio* specie; cross-reactive immune response against several *Vibrio* species including *V. parahaemolyticus*, *V. vulnificus* and *V. alginolyticus* were induced in vaccination mice [39]. Peng et al. reported that OMPs from *Vibrio parahaemolyticus* were similar to those in *P. fluorescens* and *A. hydrophila*, which could induce protective immunity against not only *Vibrio parahaemolyticus* but also *Pseudomonas fluorescens* and *A. hydrophila* in vivo [40]. Wang et al. showed that OMPs from *E. coli* were highly conserved and had cross-reaction to different Gram-negative bacteria in vitro; they indicated that these OMPs had the potential to be developed into vaccines against *Escherichia*, *Shigella* and *Salmonella* [14,15,16]. Based on previous studies and the high conserved property of OmpA, BamA and OmpC from *E. coli* O78 in the current work, the cross-reaction of these three OMPs was characterized. The whole-cell iELISA assay showed that the antisera from mice immunized with these OMPs exhibited high affinity to multiple strains including *Escherichia*, *Shigella* and *Salmonella* in vitro (Figure 3c), which was consistent with the homological analysis and previous reports [14,15,16], indicating that the OMPs in this work also had the potential to be developed into vaccines against multiple Gram-negative bacteria. However, the cross-protection of OMPs against Gram-negative bacteria in vivo needs further study.

In this work, rOmpA, rBamA and rOmpC from *E. coli* O78 (CVCC CAU0768) were separately mixed with Freund’s adjuvant in a volume ratio of 1:1, and the antibody titer of antisera was 1:32,805,000, 1:6,561,000 and 1:1,701,000, respectively; the antibody titer of the PBS-adjuvant group was 1:171,000 (Figure 3a). Previous studies showed that rOmpA, rBamA and rOmpC from *E. coli* CVCC 1515 were separately mixed with Freund’s adjuvant in a volume ratio of 3:1, the antibody titer of antisera from immunized mice was 1:642,000, 1:736,000 and 1:240,000, respectively, and the antibody titer of the PBS-adjuvant group was lower than 1:100 [14,15,16]. The above results indicated that at the same injection volume, the proportion of Freund’s adjuvant had a considerable influence on the level of the immune response; the antibody titer in antisera may increase with the proportion of Freund’s adjuvant. In addition, the nonspecific immune response of the PBS-adjuvant group was also found in the assays of total IgG level detection (Figure 4). Studies have shown that Freund’s adjuvant was one of the most effective adjuvants for inducing high antibody titer and cell-mediated immunity (Th1) [41,42]. The previous report indicated that the function of complete Freund’s adjuvant (CFA) may be sufficient to prevent group B *Coxsackieviruses* infection, and the disease resistance ability of CFA may be attributed to the adjuvanticity of *M. tuberculosis* [43]. Therefore, the nonspecific immune response in the PBS-adjuvant group in this work may also be related to the presence of the cell wall component of *M. tuberculosis* in the CFA. Studies showed that the type of adjuvant, the mixture ratio of antigen and adjuvant, injection dose and administration mode all have a non-negligible influence on the generation of the immune response [44,45], which need to be further explored.

Previous reports showed that rOmpA from several Gram-negative bacteria including *Escherichia*, *Shigella*, *Proteus* and *Edwardsiella* was an ideal antigen and could trigger significant humoral and/or cell-mediated immunity in inoculated animals [15,17,19,21]; especially, Pore et al. indicated rOmpA showed a competency to coordinate not only adaptive but also innate responses immune in mice [17]. In the current work, compared with rBamA and rOmpC, rOmpA was the most efficient antigen with more significant humoral immunity in inoculated mice, indicating rOmpA from *E. coli* O78 was also an ideal antigen that could trigger significant protective humoral immunity in inoculated animal, while the cell-mediated immunity and innate immune responses needed a further study. Moreover, in vitro and in vivo assays showed that mice immunized with rOmpA in combination with the other two proteins were detected with the highest antibody titer (Figure 3a), the highest affinity to multiple Gram-negative bacteria (Figure 3c), the highest total IgG level (Figure 4), the most significant opsonization on macrophages (Figure 5), the highest survival rate of homologous strain (*E. coli* O78) challenge (Figure 6a) and the most effective anti-infection effect of heterologous strain (*E. coli* O157) challenge (Figure 6b), indicating that the methods of combination immunization could increase the abundance of antigens, thus effectively promoting the humoral immunity with more production of functional antibodies against not only homologous strain infection but also heterologous strain infection in mice. *E. coli* O157 (CICC 21530) belongs to EHEC of IPEC, while *E. coli* O78 (CVCC CAU0768) belongs to APEC of ExPEC [46,47,48]; there was a high identity between their outer membrane proteins according to the homology analysis (Figure S1), so the proteins from *E. coli* O78 (CVCC CAU0768) also had a cross-protective reaction against the *E. coli* O157 infection in vivo. Meanwhile, although most in vitro experiments showed that rOmpC and rBamA also stimulated a considerable immune response in mice; the protection effect of bacterial challenge in vivo was less than satisfactory compared with the PBS-adjuvant group. It has been shown that IgG1 antibody was elicited by Th2 lymphocytes, while antibodies of IgG2a, IgG2b and IgG3 were elicited by Th1 lymphocytes; IgG and IgM mainly exist in blood and play important role in antibacterial, antiviral, neutralizing toxins, etc. [49,50,51]. In this work, various antibody isotypes including IgG1, IgG2a, IgG2b, and IgM were detected (Figure 3b) in antisera, which may be a vital reason for the immunized mice to resist the challenge of both homologous and heterologous strain.

In conclusion, the high conserved property of OmpA, BamA and OmpC from *E. coli* O78 (CVCC CAU0768) indicated that they could be developed into universal antigens. In vitro assays showed that antisera from immunized mice had a high affinity to multiple Gram-negative bacteria; in vivo challenge showed that mice immunized with these recombinant proteins could resist not only homologous strains but also heterologous strains. rOmpA was characterized as the most efficient antigen in this work, which provided a basis for the selection of the most effective antigen in the future. Furthermore, it was found that a stronger immune response in mice could be triggered when immunized with several proteins in combination, which provided the reference for the construction of novel antigens with higher immunogenicity. However, there are still some limitations in this work; the cross-protection of OMPs against Gram-negative bacteria in vivo and more immune-related indexes are needed to be detected to further explore the immune mechanism of OMPs.

## Data Availability

All datasets generated for this study are included in the article/Supplementary Material.

## Supplementary Materials

The following supporting information can be downloaded at: https://www.mdpi.com/article/10.3390/microorganisms10050982/s1, Figure S1: Phylogenetic trees of OmpA (a), BamA (b) and OmpC (c) in *Escherichia*, *Shigella*, *Salmonella* and *Klebsiella* constructed by MEGA; Table S1: Significance analysis for fecal shedding between the recombinant protein immunized group and the PBS-adjuvant group.
