# Supplementary material for: The Outer Membrane Proteins and Their Synergy Triggered the Protective Effects against Pathogenic Escherichia coli
Source: Microorganisms. 2022 May 8;10(5):982. doi: 10.3390/microorganisms10050982 (PMC9143122; doi:10.3390/microorganisms10050982)
Supplement: Supplementary file 1 [file microorganisms-10-00982-s001.zip › microorganisms-1701645-supplementary.pdf]

# Supplementary Information

a

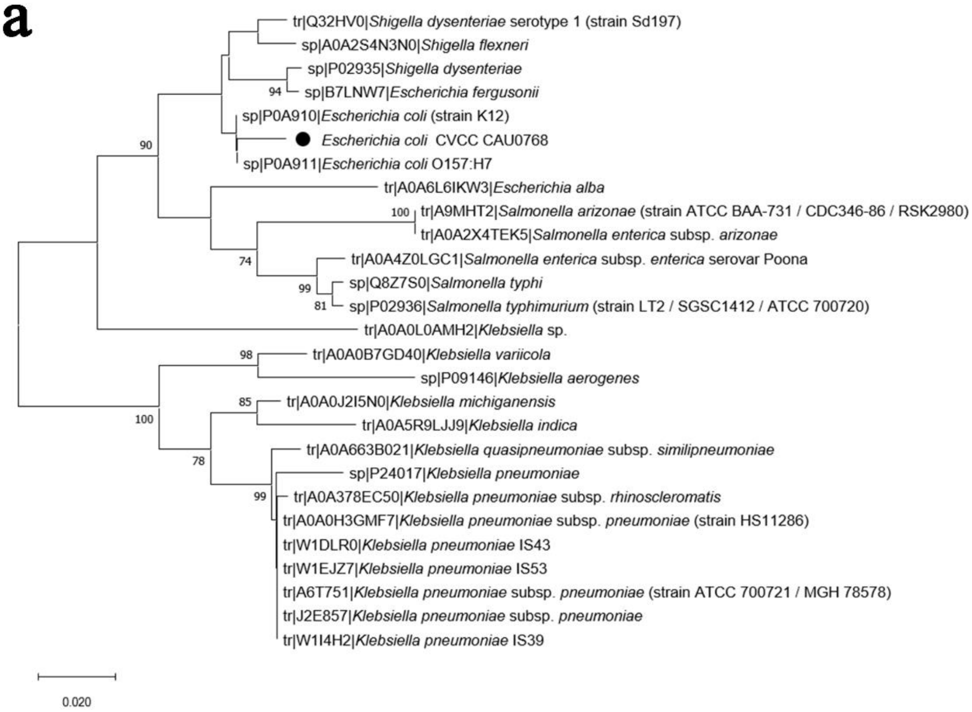

b

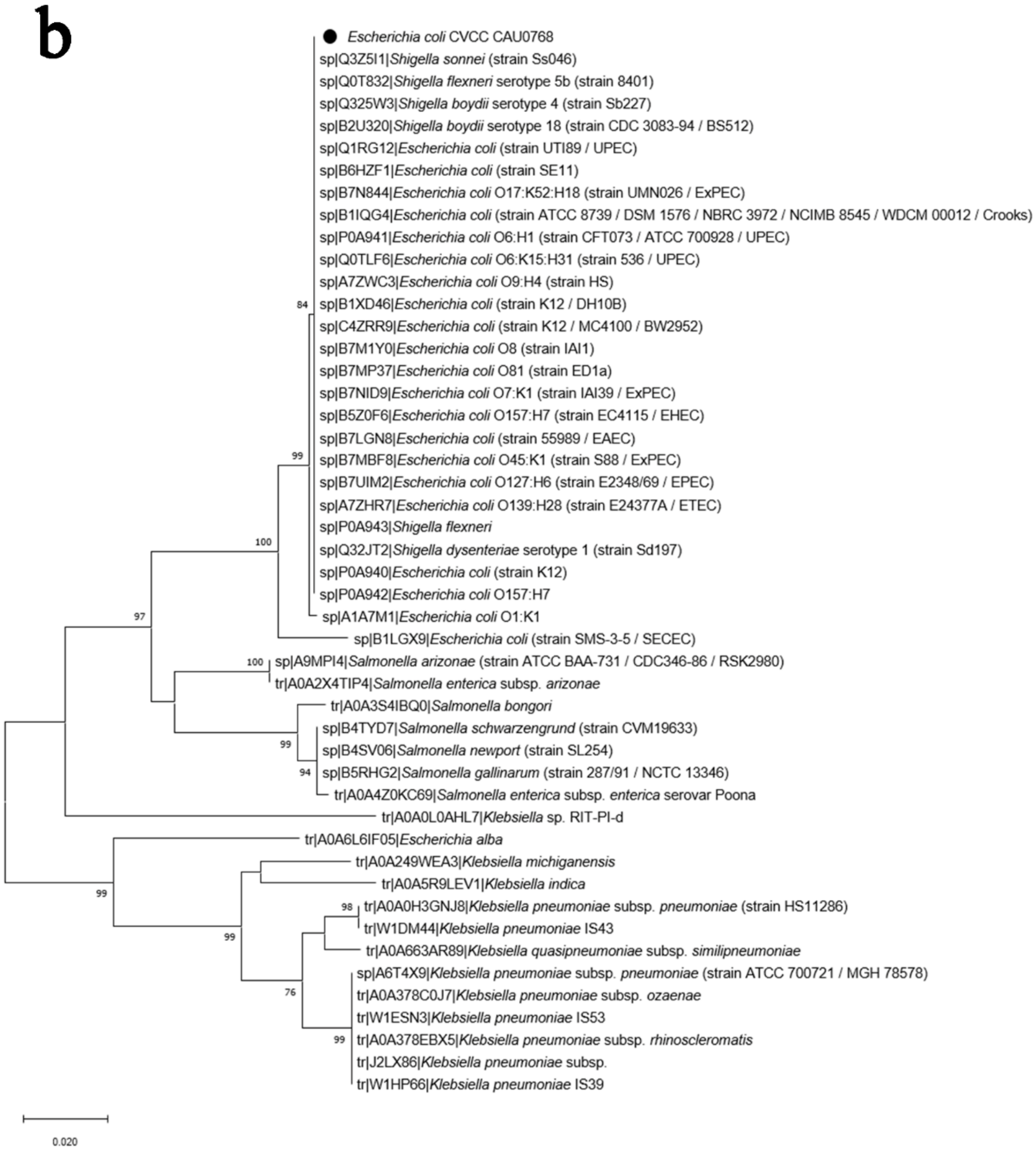

Escherichia coli CVCC CAU0768
sp|Q3Z5I1|Shigella sonnei (strain Ss046)
sp|Q0T832|Shigella flexneri serotype 5b (strain 8401)
sp|Q325W3|Shigella boydii serotype 4 (strain Sb227)
sp|B2U320|Shigella boydii serotype 18 (strain CDC 3083-94 / BS512)
sp|Q1RG12|Escherichia coli (strain UTI89 / UPEC)
sp|B6HZF1|Escherichia coli (strain SE11)
sp|B7N844|Escherichia coli O17:K52:H18 (strain UMN026 / ExPEC)
sp|B1IQG4|Escherichia coli (strain ATCC 8739 / DSM 1576 / NBRC 3972 / NCIMB 8545 / WDCM 00012 / Crooks)
sp|P0A941|Escherichia coli O6:H1 (strain CFT073 / ATCC 700928 / UPEC)
sp|Q0TLF6|Escherichia coli O6:K15:H31 (strain 536 / UPEC)
sp|A7ZWC3|Escherichia coli O9:H4 (strain HS)
sp|B1XD46|Escherichia coli (strain K12 / DH10B)
sp|C4ZRR9|Escherichia coli (strain K12 / MC4100 / BW2952)
sp|B7M1Y0|Escherichia coli O8 (strain IAI1)
sp|B7MP37|Escherichia coli O81 (strain ED1a)
sp|B7NID9|Escherichia coli O7:K1 (strain IAI39 / ExPEC)
sp|B5Z0F6|Escherichia coli O157:H7 (strain EC4115 / EHEC)
sp|B7LGN8|Escherichia coli (strain 55989 / EAEC)
sp|B7MBF8|Escherichia coli O45:K1 (strain S88 / ExPEC)
sp|B7UIM2|Escherichia coli O127:H6 (strain E2348/69 / EPEC)
sp|A7ZHR7|Escherichia coli O139:H28 (strain E24377A / ETEC)
sp|P0A943|Shigella flexneri
sp|Q32JT2|Shigella dysenteriae serotype 1 (strain Sd197)
sp|P0A940|Escherichia coli (strain K12)
sp|P0A942|Escherichia coli O157:H7
sp|A1A7M1|Escherichia coli O1:K1
sp|B1LGX9|Escherichia coli (strain SMS-3-5 / SECEC)
sp|A9MPI4|Salmonella arizonae (strain ATCC BAA-731 / CDC346-86 / RSK2980)
tr|A0A2X4TIP4|Salmonella enterica subsp. arizonae
tr|A0A3S4IBQ0|Salmonella bongori
sp|B4TYD7|Salmonella schwarzengrund (strain CVM19633)
sp|B4SV06|Salmonella newport (strain SL254)
sp|B5RHG2|Salmonella gallinarum (strain 287/91 / NCTC 13346)
tr|A0A4Z0KC69|Salmonella enterica subsp. enterica serovar Poona
tr|A0A0L0AHL7|Klebsiella sp. RIT-PI-d
tr|A0A6L6IF05|Escherichia alba
tr|A0A249WEA3|Klebsiella michiganensis
tr|A0A5R9LEV1|Klebsiella indica
tr|A0A0H3GNJ8|Klebsiella pneumoniae subsp. pneumoniae (strain HS11286)
tr|W1DM44|Klebsiella pneumoniae IS43
tr|A0A663AR89|Klebsiella quasipneumoniae subsp. similipneumoniae
sp|A6T4X9|Klebsiella pneumoniae subsp. pneumoniae (strain ATCC 700721 / MGH 78578)
tr|A0A378C0J7|Klebsiella pneumoniae subsp. ozaenae
tr|W1ESN3|Klebsiella pneumoniae IS53
tr|A0A378EBX5|Klebsiella pneumoniae subsp. rhinoscleromatis
tr|J2LX86|Klebsiella pneumoniae subsp.
tr|W1HP66|Klebsiella pneumoniae IS39
84
99
100
97
100
99
94
99
98
99
76
99
0.020

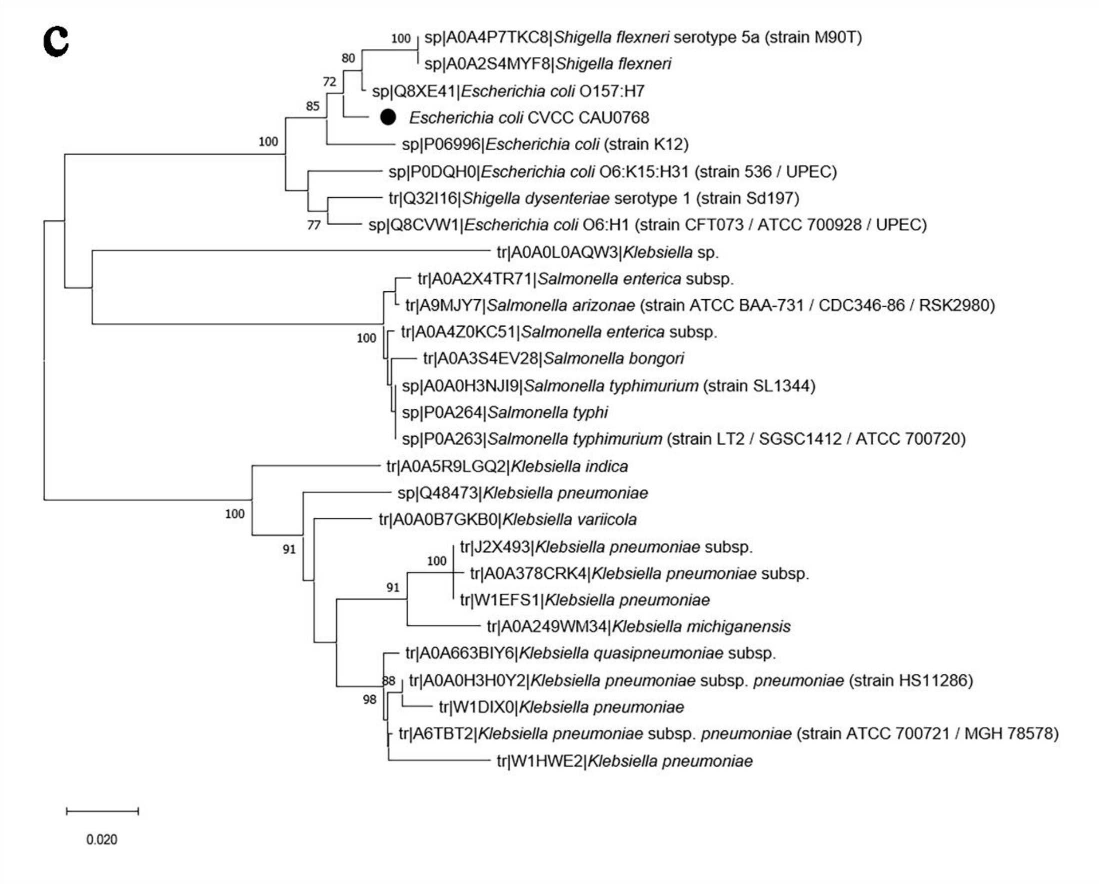


**Figure S1.** Phylogenetic trees of OmpA (**a**), BamA (**b**) and OmpC (**c**) in *Escherichia*, *Shigella*, *Salmonella* and *Klebsiella* constructed by MEGA.

**Table S1.** Significance analysis for fecal shedding between the recombinant protein immunized group and the PBS-adjuvant group.

| Groups | Days post-infection | | | | | | |
|---|---|---|---|---|---|---|---|
| | **1d** | **2d** | **3d** | **4d** | **5d** | **6d** | **7d** |
| **rOmpA-adjuvant** | ns | ns | ns | *** | ns | *** | ** |
| **rBamA-adjuvant** | ns | ns | ns | *** | ns | *** | ns |
| **rOmpC-adjuvant** | * | ns | ns | *** | ns | *** | ** |
| **rOmpA+rBamA-adjuvant** | ns | ns | * | *** | * | *** | ** |
| **rOmpA+rOmpC-adjuvant** | ** | ns | * | *** | ** | *** | ** |
| **rBamA+rOmpC-adjuvant** | ns | ns | ns | *** | ns | **** | *** |
| **rOmpA+rBamA+rOmpC-adjuvant** | ns | ns | * | ** | * | *** | * |

Note: "*, **, *** and ****" Indicates a significant difference between the recombinant protein immunized group and the PBS-adjuvant group (*$P \leq 0.05$, **$P \leq 0.01$, ***$P \leq 0.001$, ****$P \leq 0.0001$). "ns" Indicates that there is no significant difference between the recombinant protein immunized group and the PBS-adjuvant group.
